# Supplementary material for: Caregiver mental health and school-aged children’s academic and socioemotional outcomes: Examining associations and mediators in Northern Ghana
Source: PLOS Glob Public Health. 2024 Sep 13;4(9):e0003724. doi: 10.1371/journal.pgph.0003724 (PMC11398656; doi:10.1371/journal.pgph.0003724)
Supplement: Attachment — Submitted filename: Response to reviewers_R1.docx [file pgph.0003724.s001.docx]

**Reviewer #1**

In their paper, “Caregiver mental health and school-aged children’s academic and socioemotional outcomes: Examining associations and mediators in Northern Ghana”, the authors utilized a sample of 4714 children in three rural regions of northern Ghana and employed descriptive statistics, linear regressions, and SEM analyses. The primary findings are that the mechanisms through which caregiver mental health is associated with children’s developmental outcomes in Ghana, as well as how mechanisms differ as a function of caregivers’ educational attainment. I believe that this study is worthy of publication in the PLOS Global Public Health with the following considerations.

Response: Thank you for your review of our manuscript and your supportive feedback. Please see below for our point-by-point responses to your comments.

GENERAL
Comment 1. The paper is well written. But, I did see several typos. Therefore, I suggest that the authors engaged a professional editor to make sure the manuscript is as error-free as possible.

Response 1. We have conscientiously reviewed the manuscript to address all typos and grammatical mistakes.

INTRODUCTION AND LITERATURE REVIEW

Comment 2. Expand the literature review to include more international studies. This can help contextualize the findings within a global framework and highlight the relevance of the study across different cultural settings.

Response 2. We have expanded the literature review to include more studies from different country contexts to better contextualize the findings within the global literature.

- *Pages 6-7, lines 114-140 (****in document with tracked changes****)*: “It is also important to consider caregivers’ mental health given strong evidence of its consistent negative associations with children’s academic and socioemotional outcomes in HIC and LMIC contexts (Gelaye et al., 2016; Rogers et al., 2020). For example, data from various countries, including Brazil, Canada, South Africa, and the UK, consistently show that children of mothers who experience depression and anxiety are more likely to experience mental health problems such as depression, anxiety, and suicidal ideation and attempt in childhood and adolescence (Ahun et al., 2017; Gonçalves et al., 2016; Netsi et al., 2018; Orri et al., 2021; Pearson et al., 2013; Psychogiou et al., 2021). Maternal depression and anxiety are also consistently associated with poorer child and adolescent cognitive/academic outcomes across various contexts (Rogers et al., 2020). Fewer studies have examined the association of paternal mental health with child and adolescent outcomes, but a recent umbrella review – consisting primarily of studies in HICs – suggests that children of fathers who experience depression and anxiety are more likely to experience mental health problems in childhood and adolescence (Scarlett et al., 2023). Even fewer studies have focused on academic outcomes, with two studies (Norway and USA) reporting language difficulties in children whose fathers experience depression (Fredriksen et al., 2019; Malin et al., 2012).

Emerging research from HICs suggests that different dimensions of caregiver engagement mediate the association between caregiver – primarily maternal – mental health and children’s developmental outcomes (Goodman et al., 2020). Yet, there is very little research from LMICs, particularly in West African contexts (Herba et al., 2016). The handful of studies conducted in LMICs (i.e., Bangladesh, Ghana, Liberia, South Africa, Vietnam) have identified discipline, positive parent-child interactions, parenting self-efficacy, maternal intrusiveness and coerciveness, and maternal responsiveness as significant mediators of the associations of caregiver mental health with children’s cognitive/academic and socioemotional outcomes (Black et al., 2007; Huang et al., 2018; Johnson et al., 2022; Tomlinson et al., 2005; Tran et al., 2014). Further research is needed to better understand why and for whom caregiver mental health problems increase the risk of poorer child outcomes.”

Added references

Ahun, M. N., Geoffroy, M. C., Herba, C. M., Brendgen, M., Seguin, J. R., Sutter-Dallay, A. L., . .

. Cote, S. M. (2017). Timing and Chronicity of Maternal Depression Symptoms and

Children's Verbal Abilities. *Journal of Pediatrics, 190*, 251-257.

Black, M. M., Baqui, A. H., Zaman, K., McNary, S. W., Le, K., Arifeen, S. E., ... & Black, R. E.

(2007). Depressive symptoms among rural Bangladeshi mothers: implications for infant

development. *Journal of Child Psychology and Psychiatry*, *48*(8), 764-772.

Fredriksen, E., von Soest, T., Smith, L., & Moe, V. (2019). Parenting stress plays a mediating

role in the prediction of early child development from both parents’ perinatal depressive

symptoms. *Journal of Abnormal Child Psychology, 47*(1), 149-164.

Gonçalves, H., Pearson, R. M., Horta, B. L., González-Chica, D. A., Castilho, E., Damiani, M.,

... & Victora, C. G. (2016). Maternal depression and anxiety predicts the pattern of

offspring symptoms during their transition to adulthood. *Psychological Medicine, 46*(2),

415-424.

Malin, J. L., Karberg, E., Cabrera, N. J., Rowe, M., Cristaforo, T., & Tamis-LeMonda, C. S.

(2012). Father-toddler communication in low-income families: The role of paternal

education and depressive symptoms. *Fam Sci, 3*(3-4), 155-163.

Netsi, E., Pearson, R. M., Murray, L., Cooper, P., Craske, M. G., & Stein, A. (2018). Association

of Persistent and Severe Postnatal Depression With Child Outcomes. *JAMA Psychiatry,*

*75*(3), 247-253. doi:https://doi.org/10.1001/jamapsychiatry.2017.4363

Orri, M., Besharati, S., Ahun, M. N., & Richter, L. M. (2021). Analysis of maternal postnatal

depression, socioeconomic factors, and offspring internalizing symptoms in a

longitudinal cohort in South Africa. *JAMA Network Open*, *4*(8), e2121667-e2121667

Pearson, R. M., Evans, J., Kounali, D., Lewis, G., Heron, J., Ramchandani, P. G., ... & Stein, A.

(2013). Maternal depression during pregnancy and the postnatal period: risks and

possible mechanisms for offspring depression at age 18 years. *JAMA Psychiatry*, *70*(12),

1312-1319.

Psychogiou, L., Ahun, M. N., Boivin, M., Tremblay, R. E., Orri, M., & Côté, S. M. (2022). The

mediating role of adolescents’ loneliness and social withdrawal in the association

between maternal depressive symptoms and suicidality in adolescence: A 20-year

population-based study. *Development and Psychopathology*, *34*(3), 1045-1053.

Scarlett, H., Moirangthem, S., & van der Waerden, J. (2023). The impact of paternal mental

illness on child development: an umbrella review of systematic reviews and meta

analyses. *European Child & Adolescent Psychiatry*, 1-14

Tomlinson, M., Cooper, P., & Murray, L. (2005). The Mother–Infant Relationship and Infant

Attachment in a South African Peri-Urban Settlement. *Child Development*, *76*(5), 1044-1054.

Tran, T. D., Biggs, B.-A., Tran, T., Simpson, J. A., de Mello, M. C., Hanieh, S., Nguyen, T. T.,

Dwyer, T., & Fisher, J. (2014). Perinatal common mental disorders among women and

the social and emotional development of their infants in rural Vietnam. *Journal of*

*Affective Disorders*, *160*, 104–112.

METHODS

Comment 3. While the study uses established scales, more information on their validity and reliability in the specific context is needed. The approaches to dealing with missing data should be reported.

Response 3. We have provided more information on the validity and reliability on the scales used in our study. For parsimony’s sake, we only include some examples of these revisions here. We reported our approach to dealing with missing data in the methods section.

- *Page 11, lines 236-244:* “Children’s socioemotional skills were assessed with a caregiver-report and a direct assessment measure. Caregivers reported the extent to which each child exhibited socioemotional difficulties in the last six months using the Strengths and Difficulties Questionnaire ($\alpha$ = 0.71), which has been validated in the Ghanaian context (Goodman, 1997; Hoosen et al., 2018). In addition, children’s prosocial conflict resolution skills and relationships (i.e., how much children draw on relational support from caregivers and other community members in challenging times) ($\alpha$ = 0.40) were assessed using the International Social-Emotional Learning Assessment, a tool specifically developed for use in low-resource contexts and previously validated in other African countries (D’Sa & Krupar, 2021; Jones et al., 2020).”
- *Page 15, lines 314-315*: “Missing data were handled using the full information maximum likelihood.”

Added references

Hoosen, N., Davids, E. L., de Vries, P. J., & Shung-King, M. (2018). The Strengths and Difficulties

Questionnaire (SDQ) in Africa: a scoping review of its application and validation. *Child*

*And Adolescent Psychiatry And Mental Health, 12*, 1-39.

Jones, S. M., Bailey, R., Temko, S., Donaher, M., Raisch, N., & Ramirez, T. (2020). SEL and PSS

measurement and assessment tools in education in emergencies: Identifying, analyzing,

and mapping tools to global guidance documents. Inter-Agency Network for Education in

Emergencies (INEE).

DISCUSSION

Comment 4. The discussion does not sufficiently cover the practical applications of the findings. There need to be more discussions in this section. To as great as extent possible, make sure all of the explanations are also clarifying or expanding on the literature cited in the Introduction and Literature Review section.

Response 4. We have revised the discussion to include reflections on the practical applications of the findings and to expand on the literature cited in the introduction and literature review section. For parsimony’s sake, we only include some examples of these revisions here.

- *Page 19, lines 406-415*: “Our results – from a sample of male and female caregivers – replicate and extend findings documenting the association of female caregivers’ mental health with child and adolescent academic and socioemotional outcomes across various contexts. Additionally, this is one of the few studies to demonstrate that male caregivers’ mental health is significantly associated with poorer academic and socioemotional outcomes in an LMIC context. These findings suggest that both maternal and paternal mental health play a role in children’s development and underscore the importance of including both female and male caregivers in interventions to support family wellbeing and child development. Our findings also suggest that targeting caregiver engagement in education in families where caregivers have no formal education could be a fruitful way to improve academic outcomes for children and adolescents.”
- *Pages 25-26, lines 555-567*: “This study sheds light on the mechanisms through which caregiver mental health is associated with children’s development in Ghana, as well as how mechanisms differ as a function of caregivers’ exposure to formal education. Our findings suggest that addressing caregiver mental health could be a viable component of interventions targeting caregiver engagement in children’s education, particularly for families with no formal education. However, the strategies and interventions to optimize caregiver engagement and child outcomes must be culturally and contextually driven (Appiah, 2022) and tailored to match caregivers’ educational backgrounds, capacities, and circumstances if they are to be effective. Leveraging longitudinal and qualitative methodologies, such co-created, context-specific, tailored approaches can contribute to reducing the mental health burden of Ghanaian parents and increasing their children’s developmental potential.”

In summary, then, I am supportive of this manuscript moving forward towards publication. I recommend “accept with major revisions”. I would be more than happy to review any revised manuscript, if the editor needs may help.

**Reviewer #2**

Comment 1. It is recommended to add a literature review in the introduction section. That is, what research currently exists on the relationship between caregiver depression and child development. This will help readers understand the current research status.

Response 1. We have added more information on the current association between caregiver depression and child development to the literature review.

- *Pages 6-7, lines 114-140 (****in document with tracked changes****)*: “It is also important to consider caregivers’ mental health given strong evidence of its consistent negative associations with children’s academic and socioemotional outcomes in HIC and LMIC contexts (Gelaye et al., 2016; Rogers et al., 2020). For example, data from various countries, including Brazil, Canada, South Africa, and the UK, consistently show that children of mothers who experience depression and anxiety are more likely to experience mental health problems such as depression, anxiety, and suicidal ideation and attempt in childhood and adolescence (Ahun et al., 2017; Gonçalves et al., 2016; Netsi et al., 2018; Orri et al., 2021; Pearson et al., 2013; Psychogiou et al., 2021). Maternal depression and anxiety are also consistently associated with poorer child and adolescent cognitive/academic outcomes across various contexts (Rogers et al., 2020). Fewer studies have examined the association of paternal mental health with child and adolescent outcomes, but a recent umbrella review – consisting primarily of studies in HICs – suggests that children of fathers who experience depression and anxiety are more likely to experience mental health problems in childhood and adolescence (Scarlett et al., 2023). Even fewer studies have focused on academic outcomes, with two studies (Norway and USA) reporting language difficulties in children whose fathers experience depression (Fredriksen et al., 2019; Malin et al., 2012).

Emerging research from HICs suggests that different dimensions of caregiver engagement mediate the association between caregiver – primarily maternal – mental health and children’s developmental outcomes (Goodman et al., 2020). Yet, there is very little research from LMICs, particularly in West African contexts (Herba et al., 2016). The handful of studies conducted in LMICs (i.e., Bangladesh, Ghana, Liberia, South Africa, Vietnam) have identified discipline, positive parent-child interactions, parenting self-efficacy, maternal intrusiveness and coerciveness, and maternal responsiveness as significant mediators of the associations of caregiver mental health with children’s cognitive/academic and socioemotional outcomes (Black et al., 2007; Huang et al., 2018; Johnson et al., 2022; Tomlinson et al., 2005; Tran et al., 2014). Further research is needed to better understand why and for whom caregiver mental health problems increase the risk of poorer child outcomes.”

Added references

Ahun, M. N., Geoffroy, M. C., Herba, C. M., Brendgen, M., Seguin, J. R., Sutter-Dallay, A. L., . .

. Cote, S. M. (2017). Timing and Chronicity of Maternal Depression Symptoms and

Children's Verbal Abilities. *Journal of Pediatrics, 190*, 251-257.

Black, M. M., Baqui, A. H., Zaman, K., McNary, S. W., Le, K., Arifeen, S. E., ... & Black, R. E.

(2007). Depressive symptoms among rural Bangladeshi mothers: implications for infant

development. *Journal of Child Psychology and Psychiatry*, *48*(8), 764-772.

Fredriksen, E., von Soest, T., Smith, L., & Moe, V. (2019). Parenting stress plays a mediating

role in the prediction of early child development from both parents’ perinatal depressive

symptoms. *Journal of Abnormal Child Psychology, 47*(1), 149-164.

Gonçalves, H., Pearson, R. M., Horta, B. L., González-Chica, D. A., Castilho, E., Damiani, M.,

... & Victora, C. G. (2016). Maternal depression and anxiety predicts the pattern of

offspring symptoms during their transition to adulthood. *Psychological Medicine, 46*(2),

415-424.

Malin, J. L., Karberg, E., Cabrera, N. J., Rowe, M., Cristaforo, T., & Tamis-LeMonda, C. S.

(2012). Father-toddler communication in low-income families: The role of paternal

education and depressive symptoms. *Fam Sci, 3*(3-4), 155-163.

Netsi, E., Pearson, R. M., Murray, L., Cooper, P., Craske, M. G., & Stein, A. (2018). Association

of Persistent and Severe Postnatal Depression With Child Outcomes. *JAMA Psychiatry,*

*75*(3), 247-253. doi:https://doi.org/10.1001/jamapsychiatry.2017.4363

Orri, M., Besharati, S., Ahun, M. N., & Richter, L. M. (2021). Analysis of maternal postnatal

depression, socioeconomic factors, and offspring internalizing symptoms in a

longitudinal cohort in South Africa. *JAMA Network Open*, *4*(8), e2121667-e2121667

Pearson, R. M., Evans, J., Kounali, D., Lewis, G., Heron, J., Ramchandani, P. G., ... & Stein, A.

(2013). Maternal depression during pregnancy and the postnatal period: risks and

possible mechanisms for offspring depression at age 18 years. *JAMA Psychiatry*, *70*(12),

1312-1319.

Psychogiou, L., Ahun, M. N., Boivin, M., Tremblay, R. E., Orri, M., & Côté, S. M. (2022). The

mediating role of adolescents’ loneliness and social withdrawal in the association

between maternal depressive symptoms and suicidality in adolescence: A 20-year

population-based study. *Development and Psychopathology*, *34*(3), 1045-1053.

Scarlett, H., Moirangthem, S., & van der Waerden, J. (2023). The impact of paternal mental

illness on child development: an umbrella review of systematic reviews and meta

analyses. *European Child & Adolescent Psychiatry*, 1-14

Tomlinson, M., Cooper, P., & Murray, L. (2005). The Mother–Infant Relationship and Infant

Attachment in a South African Peri-Urban Settlement. *Child Development*, *76*(5), 1044-1054.

Tran, T. D., Biggs, B.-A., Tran, T., Simpson, J. A., de Mello, M. C., Hanieh, S., Nguyen, T. T.,

Dwyer, T., & Fisher, J. (2014). Perinatal common mental disorders among women and

the social and emotional development of their infants in rural Vietnam. *Journal of*

*Affective Disorders*, *160*, 104–112.

Comment 2. Although this study is a randomized intervention experiment, the sampling still has a self-selection bias. If this study is a randomized intervention experiment, it is necessary to show the balance test of the basic data. Check whether the baseline personal characteristics and outcome variables are balanced. And inform us if the intervention group and the control group were selected through random assignment.

Response 2. The balance test of the basic data is reported in the working paper that presents the primary results of the intervention (Aurino & Wolf, 2024). The test found baseline equivalence across the treatment and control groups for most covariates, except a few (i.e., ownership of television, caregiver low educational aspirations for boys, living in the North East region). We have included this information in the Methods section, along with an explicit statement that the intervention and control groups were assigned through random assignment. We account for differences in the outcome variables across the treatment and control groups by controlling for intervention arm in the mediation models.

- *Page 8, lines 172-177*: “After consenting to participate, families were randomly assigned to one of the treatment conditions or the control group. The treatment and control groups were statistically equivalent across a broad range of baseline characteristics. The only significant differences were that a greater number of control families owned a television, and treatment families were more likely to live in the North East region and had low educational aspirations for male children (Aurino & Wolf, 2024).”
- *Page 13, lines 281-283*: “The mediation models controlled for intervention status (control vs treatment arms), and midline levels of the mediators and dependent variables.”

Reference

Aurino, E., & Wolf, S. (2024). A ‘smart buy’ for all? Unequal and unintended consequences of a

messaging program for child education. UB Economics–Working Papers, 2024 E24/461.

<https://diposit.ub.edu/dspace/handle/2445/205763>

Comment 3. It is recommended that the author state the reliability and validity of all the scales used. This will allow readers to understand the effectiveness of these scales.

Response 3. We have provided more information on the validity and reliability on the scales used in our study. For parsimony’s sake, we only include some examples of these revisions here.

- *Page 11, lines 236-244:* “Children’s socioemotional skills were assessed with a caregiver-report and a direct assessment measure. Caregivers reported the extent to which each child exhibited socioemotional difficulties in the last six months using the Strengths and Difficulties Questionnaire ($\alpha$ = 0.71), which has been validated in the Ghanaian context (Goodman, 1997; Hoosen et al., 2018). In addition, children’s prosocial conflict resolution skills and relationships (i.e., how much children draw on relational support from caregivers and other community members in challenging times) ($\alpha$ = 0.40) were assessed using the International Social-Emotional Learning Assessment, a tool specifically developed for use in low-resource contexts and previously validated in other African countries (D’Sa & Krupar, 2021; Jones et al., 2020).”

Added references

Hoosen, N., Davids, E. L., de Vries, P. J., & Shung-King, M. (2018). The Strengths and Difficulties

Questionnaire (SDQ) in Africa: a scoping review of its application and validation. *Child*

*And Adolescent Psychiatry And Mental Health, 12*, 1-39.

Jones, S. M., Bailey, R., Temko, S., Donaher, M., Raisch, N., & Ramirez, T. (2020). SEL and PSS

measurement and assessment tools in education in emergencies: Identifying, analyzing,

and mapping tools to global guidance documents. Inter-Agency Network for Education in

Emergencies (INEE).

Comment 4. This study mainly analyzes the relationship between caregiver mental health and child development. There is a strong endogeneity issue, such as the bidirectional causality between caregiver mental health and child development. This study mainly analyzes the direct and indirect effects between the two through structural models. Therefore, it is recommended that the author conduct an endogeneity analysis.

Response 4. We thank the reviewer for their comment. We agree that there may be endogeneity in the relation between caregiver psychological distress and child development, with unobserved factors being correlated with both independent and dependent variables. For this reason, we are careful in not making any causal claims. We have added a sentence in the text saying that we do not claim any causation in our statistical analysis, and that all the relations we uncover are associations. We also acknowledge the cross-sectional nature of our data in the limitations. We were not sure what the reviewer meant by ‘endogeneity analysis’, but we hope that these statements address the reviewer’s comment.

- *Pages 13-14, lines 289-291*: “The term ‘effect’ is used in line with SEM nomenclature to describe associations between variables (Kline, 2015). Given the cross-sectional nature of these data, we acknowledge that these ‘effects’ are not causal.”
- *Page 24, lines 529-532*: “Firstly, although our analyses are based on strong empirical evidence on the nature of associations between caregiver mental health, caregiver engagement, and child outcomes, the use of cross-sectional data precludes us from making any claims about the causality or directionality of the reported findings.”

Reference

Kline, R. B. (2015). *Principles and practice of structural equation modeling*. Guilford

publications.

Comment 5. It is recommended that the author test whether there are significant differences in child outcome variables and personal characteristics between caregivers who received education and those who did not in Table 1. It is also suggested that the author first test the average effect and then conduct separate analyses for the groups where caregivers received education and those who did not.

Response 5. Thank you for this suggestion, we agree this is important given the findings. We tested for significant differences in child outcome variables and personal characteristics between caregivers who received a formal education and those who did not in Table 1. Significant differences are indicated with an asterisk as indicated in the note beneath the Table. We have included information on the specific tests used to examine differences for clarity. Regarding the reviewer’s comment on testing the average effect before conducting separate analyses, since the test of model fit indicated that the freely estimated models are a better fit to the data than the constrained model, we do not present the average estimates as these are not the best fit to the data (Kline, 2015).

- *Page 43, lines 917-918*: Table 1 footnote – “*Differences between caregivers with no formal education versus caregivers with some formal education is significant at *p<0.05* according to chi-square (for categorical variables) and ANOVA (for continuous variables) tests.”

Reference

Kline, R. B. (2015). *Principles and practice of structural equation modeling*. Guilford

publications.

**Reviewer #3**

I commend the authors for a paper that outlines the mediating role of caregiver engagement in the association between caregiver psychological distress and children’s outcomes. This study is important given that there is limited research on the underlying mechanisms of these associations in LMICs, and in particular Ghana, which is the study’s country of focus. Their results highlight the importance of using culturally sensitive approaches in early intervention programs.

Response: Thank you for your review of our manuscript and your supportive feedback. Please see below for our point-by-point responses to your comments.

My comments are as follows:

KEYWORDS

Comment 1. The authors should consider using key words that make their paper more likely to be picked up through a search. The words they have used are very generic.

Response 1. We have provided more specific keywords.

- *Page 3, lines 47-49 (****in document with tracked changes****)*: “Keywords: caregiver psychological distress; child socioemotional skills; child academic skills; caregiver engagement in education; Ghana; parenting interventions.”

INTRODUCTION

Comment 2. Line 64: Please indicate the source from which the statistics for the human capital index are obtained

Response 2. The source of this statistic is a 2018 World Bank report. We have clarified this in the manuscript.

- *Page 4, lines 69-72*:“ Ghana, a lower-middle-income country in West Africa, has a human capital index of 0.44, meaning that children born today are only expected to reach 44% of their developmental potential (World Bank, 2018).”

Added reference

World Bank. (2018). *Piecing Together the Poverty Puzzle*. Washington, DC: World Bank.

https://doi.org/10.1596/978-1-4648-1330-6

Comment 3. Line 71: It may be worthwhile to provide comparative information on global estimates in relation to mental health problems among children in Ghana.

Response 3. We have provided global estimates on the prevalence of different mental health problems to enable readers to directly compare these data with Ghanaian estimates.

- *Page 4, lines 75-79*: “Ghanaian children are also at risk of significant mental health problems, with studies reporting prevalence rates of depression (1-18%), anxiety (1-4%), and conduct problems (13%) (Addy et al., 2021; Adjorlolo, 2019; Kusi-Mensah et al., 2019) that are relatively higher than some recent global estimates: depression (1.8-2.6%), anxiety (3.4-6.5%), conduct/disruptive disorders (1.5-5.7%) (Kieling et al., 2024; Polanczyk et al., 2015).”

Comment 4. Line 73: The authors should attempt to link the idea in this paragraph to the previous one.

Response 4. We have modified the beginning of this paragraph to better link it to the previous one.

- *Page 4, lines 80-84*: “Exposure to poverty and food insecurity are known to significantly impact children’s development and mental health outcomes (Aboud & Yousafzai, 2015; Lu et al., 2016). In addition to addressing these factors, low-cost solutions targeting modifiable risk and protective factors are urgently needed to improve children’s developmental potential and subsequent health and educational outcomes (Black et al., 2021).”

Comment 5. Line 81: It is not clear what ‘it’ refers to in the statement that begins with ‘It is a key input...’.

Response 5. This sentence refers to caregiver engagement; we have clarified this in the text.

- *Page 5, lines 90-93*: “Caregiver engagement has been repeatedly shown to be associated with significant improvements in children’s academic and socioemotional outcomes (Albanese et al., 2019; Black et al., 2017; Britto et al., 2017; Kyei & Nyarko, 2024; Richter et al., 2017).”

Comment 6. Line 87: The phrase ‘negatively influence’ appears twice in the same statement which makes it cumbersome to read. The statement should be shortened to improve clarity.

Response 6. Thank you for picking this up. We have modified and re-structured this sentence into two shorter sentences to improve clarity.

- *Page 5, lines 102-106*: “The experience of mental health problems can negatively influence caregivers’ beliefs in their parenting abilities, as well as their capacity to engage in emotionally supportive and responsive interactions with their child. Caregiver mental health problems and their association with less positive parenting practices can subsequently increase the risk of worse child outcomes (Goodman et al., 2020, 2022).”

METHODS

Comment 7. The subsection on study population has information on study site, intervention, study design etc. The authors could consider having other subsections under this to help organize the section and make it more navigable for the reader.

Response 7. We have re-organized this section (*pages 7-9, lines 149-195*) to include additional subsections (study population, study site and design, intervention), making it more navigable for the reader.

Comment 8. Line 145: The ethics statement should be written chronologically in the order of obtaining ethics approval – first from the ethics body and then from the study participants. Was there any permission required at the national and subnational levels?

Response 8. We have re-written the ethics statement chronologically. The ethics body that approved this study is a Ghana-based non-governmental organization that operates across the country. We have clarified this in the manuscript.

- *Pages 9-10, lines 198-201*: “The study’s protocol was reviewed and approved by the Institutional Review Board at Innovation for Poverty Action 2020, a global non-governmental organization with an office in Ghana (#15573). Informed written consent was obtained from all adult participants and from the parent/legal guardian of all participants below 18 years of age.”

Comment 9. Line 160: I suggest that ‘We therefore...’ be the beginning of a new sentence
What was the rationale for using one SD as the cut-off?

Response 9. We have modified the sentence accordingly. We followed the widely used practice in the mental health literature (e.g., Squires, Bricker, and Twombly 2015, page 194), where one standard deviation is used as the cut-off for normally distributed data.

Reference

Squires, J., Bricker, D., & Twombly, E. (2015). *Ages and Stages Questionnaires®: Social*

*Emotional User’s Guide Second Edition.* Paul H. Brookes Publishing Co. Baltimore, USA.

Comment 10. Line 165: What does this statement mean? ‘Children’s academic skills were assessed using assessments adapted by existing scales.’

Response 10. We have modified this section to clarify that various assessments were used to measure children’s academic skills.

- *Page 10, lines 220-222*: “To accommodate the wide age range of children in the sample, three different assessments that varied the number and difficulty of items based on child age were used to measure children’s academic skills.”

Comment 11. Line 170: IDELA does not appear on the list of abbreviations. Kindly check that all abbreviations are included on the list

Response 11. We have included IDELA and all other abbreviations in the list of abbreviations at the beginning of the manuscript.

Comment 12. Line 184: The authors should indicate if the International Social-Emotional Learning Assessment has been validated in Ghana or other LMICs.

Response 12. The International Social-Emotional Learning Assessment tool has been validated in other LMICs in Africa and the Strengths and Difficulties Questionnaire has been validated in the Ghanaian context. This has now been clarified in the manuscript.

- *Page 11, lines 236-244:* “Children’s socioemotional skills were assessed with a caregiver-report and a direct assessment measure. Caregivers reported the extent to which each child exhibited socioemotional difficulties in the last six months using the Strengths and Difficulties Questionnaire ($\alpha$ = 0.71), which has been validated in the Ghanaian context (Goodman, 1997; Hoosen et al., 2018). In addition, children’s prosocial conflict resolution skills and relationships (i.e., how much children draw on relational support from caregivers and other community members in challenging times) ($\alpha$ = 0.40) were assessed using the International Social-Emotional Learning Assessment, a tool specifically developed for use in low-resource contexts and previously validated in other African countries (D’Sa & Krupar, 2021; Jones et al., 2020).”

Added references

Hoosen, N., Davids, E. L., de Vries, P. J., & Shung-King, M. (2018). The Strengths and Difficulties

Questionnaire (SDQ) in Africa: a scoping review of its application and validation. *Child*

*And Adolescent Psychiatry And Mental Health, 12*, 1-39.

Jones, S. M., Bailey, R., Temko, S., Donaher, M., Raisch, N., & Ramirez, T. (2020). SEL and PSS

measurement and assessment tools in education in emergencies: Identifying, analyzing,

and mapping tools to global guidance documents. Inter-Agency Network for Education in

Emergencies (INEE).

Comment 13. Line 239: What fit indices were considered when performing the SEM?

Response 13. We used the root mean square error of approximation and confirmatory fit index to determine model fit. We have clarified this in the methods and reported the indices in the results.

- *Pages 14-15, lines 312-314*: “Adequate model fit for the final models was considered using a root mean square error of approximation (RMSEA) of <0.06 and confirmatory fit index (CFI) of > 0.90 (Bentler, 1990; Hu & Bentler, 1999).”
- *Page 17, lines 365-367*: “In families where caregivers had no formal education, children exposed to higher levels of caregiver psychological distress had poorer literacy skills (RMSEA = 0.034, CFI = 0.979), and this association was partially mediated by caregiver engagement in education (p<0.05).”
- *Page 17, lines 370-376*: “Caregiver psychological distress was associated with higher levels of socioemotional difficulties in children (RMSEA = 0.032, CFI = 0.920), but none of the mediators explained this association. Finally, although caregiver engagement in education was significantly associated with both caregiver psychological distress and children’s numeracy skills (RMSEA = 0.035, CFI = 0.977), the indirect effect did not reach significance. None of the other hypothesized associations in the mediation models were significant. Caregiver psychological distress was not associated with children’s prosocial skills (RMSEA = 0.029, CFI = 0.955).”

Added references

Bentler, P.M., 1990. Comparative fit indexes in structural models. Psychol. Bull. 107, 238–246.

<http://dx.doi.org/10.1037/0033-2909.107.2.238>.

Hu, L. T., & Bentler, P. M. (1999). Cutoff criteria for fit indexes in covariance structure analysis:

Conventional criteria versus new alternatives. *Structural Equation Modeling: A*

*Multidisciplinary Journal, 6*(1), 1-55.

RESULTS

Comment 14. Line 256: The authors need to provide information on the ethnic groups in Ghana so that there is some context for the information provided on ethnicity. Was ethnic group one an important consideration for the analysis?

Response 14. Ethnic group was not an important consideration for the analysis; we solely provided this information to describe the ethnic make-up of our sample. We have provided some context on the Dagomba ethnic group in Ghana.

- *Page 15, lines 326-328*: “Most families are of Dagomba ethnicity (93%), which is part of the second most dominant ethnic group (Mole-Dagbani) in Ghana (Ghana Statistical Service & ICF, 2024).”

Added reference

Ghana Statistical Service (GSS) and ICF. 2024. Ghana Demographic and Health Survey 2022.

Accra, Ghana, and Rockville, Maryland, USA: GSS and ICF

Comment 15. Line 264: ‘The prevalence of elevated caregiver psychological distress was 14%, with a slightly higher – but not significantly different (2 = 1.99, p=0.157) – prevalence in caregivers who had no formal education (15%) compared to caregivers that had some formal education (13%).’ This could be stated as ‘There was no significant difference in the prevalence of elevated caregiver psychological distress among those who had no formal education (15%) and those who had some formal education (13%)’ or something along those lines.

Response 15. We have modified this sentence accordingly.

- *Page 16, lines 336-339*: “There was no significant difference ($\chi$^2^ = 1.99, *p=0.157*) in the prevalence of elevated caregiver psychological distress among caregivers who had no formal education (15%) and those who had some formal education (13%).”

Comment 16. Line 281: ‘were’ should be ‘where’ The entire paper should be checked for typographical and grammatical errors to improve quality.

Response 16. We have corrected this error and double-checked the manuscript to correct all typographical and grammatical errors and improve quality.

Comment 17. Line 291: Were the children exposed to higher levels of caregiver psychological distress or is it that they lived in households where their caregivers had higher levels of psychological distress?

Response 17. The children were exposed to higher levels of caregiver psychological distress because they were living and interacting with their caregivers, some of whom were experiencing high levels of psychological distress.

Comment 18. Like I indicated earlier, the model fit indices for the SEM should be reported within the text so that the reader can make a judgement on the outcome of the analysis

Response 18. Thank you for this comment. We have reported the model fit indices in the results (*page 17, lines 365-376*). Please see our response to comment #13.

Discussion

Comment 19. The discussion section could be improved by consistently providing possible explanations for the results presented in the paper. This has been done for some of the results and not for others.

Response 19. Thank you for this suggestion. In line with both your and reviewer 1’s comments, we have revised the discussion to provide possible explanations all of the results. For parsimony’s sake, we only include some examples of these revisions here.

- *Pages 21-22, lines 461-476*: “Overall, these findings point to the importance of understanding the context within which families live and acknowledging the role of multiple intersecting factors that significantly impact children’s development and mental health when designing interventions to improve child outcomes (Alozkan‐Sever et al., 2023; Black et al., 2021). They also underscore the importance of designing, implementing, and evaluating the impact of multi-component interventions to successfully enhance caregiver engagement and improve caregiver mental health to promote child development and family wellbeing (Goodman & Garber, 2017; Pitchik et al., 2020). A recent global systematic review of such interventions found that although they improve children’s cognitive and socioemotional outcomes, there is no meta-analytic effect on depressive symptoms in female caregivers (Al Sager et al., In Press). This is likely explained by the fact that interventions are overwhelmingly focused on improving caregiver engagement and include little content addressing caregiver mental health. To fully realize the potential of multi-component interventions to support children and their families in various contexts, we need to design programs that adequately address caregiver engagement and caregiver mental health.”
- *Page 22, lines 479-491*: “Although studies have consistently found associations between caregiver mental health – particularly depression – and parenting self-efficacy (Goodman et al., 2022), and between parenting self-efficacy and children’s developmental outcomes (Albanese et al., 2019), only a handful of studies have formally tested the mediating role of parenting self-efficacy in the caregiver mental health-child development association, most of them in HICs (Albanese et al., 2019). These few studies suggest that caregivers’ belief in their parenting abilities does explain some portion of the association between their mental health and their children’s developmental outcomes (Albanese et al., 2019; Tran et al., 2014) , but further research is needed to strengthen this evidence base. It is worth noting that these studies focused on very young children (<3 years old) and there is some evidence of a stronger association between caregiver mental health and parenting self-efficacy in the first few years post-partum (Goodman et al., 2022). It is therefore likely that we found no mediating role of parenting self-efficacy in our sample due to the older age of children (5-17 years old).”

Added reference

Al Sager, A., Goodman, S.H., Jeong, J., Bain, P., & Ahun, M.N. (*in press*). Promoting early

childhood development and parent outcomes through multi-component parenting and

parental mental health interventions: A global meta-analytic review. *The Lancet Child &*

*Adolescent Health*

Comment 20. Line 415: What other dimensions of caregiving could be considered?

Response 20. We have included suggestions of other dimensions of caregiving that could be considered in future research in this sentence.

- *Pages 24-25, lines 536-539*: “Finally, the reported associations were generally small, suggesting that other dimensions of caregiving (e.g., responsivity and sensitivity in caregiver-child interactions, disciplinary strategies) would be important to consider in future research.”
